# Six Key Advantages and Disadvantages of Working from Home in Europe during COVID-19

**DOI:** 10.3390/ijerph18041826

**Published:** 2021-02-13

**Authors:** Christine Ipsen, Marc van Veldhoven, Kathrin Kirchner, John Paulin Hansen

**Affiliations:** 1 DTU Management Department, Technical University of Denmark, 2800 Kgs. Lyngby, Denmark; chip@dtu.dk (C.I.); jpha@dtu.dk (J.P.H.); 2 Department of Human Resource Studies, Tilburg University, 5037 AB Tilburg, The Netherlands; M.J.P.M.vanVeldhoven@tilburguniversity.edu

**Keywords:** COVID-19, experiences, working from home, survey, advantages, disadvantages, knowledge work

## Abstract

The number of people working from home (WFH) increased radically during the coronavirus (COVID-19) pandemic. The purpose of this study was therefore to investigate people’s experiences of WFH during the pandemic and to identify the main factors of advantages and disadvantages of WFH. Data from 29 European countries on the experiences of knowledge workers (*N* = 5748) WFH during the early stages of lockdown (11 March to 8 May 2020) were collected. A factor analysis showed the overall distribution of people’s experiences and how the advantages and disadvantages of WFH during the early weeks of the pandemic can be grouped into six key factors. The results indicated that most people had a more positive rather than negative experience of WFH during lockdown. Three factors represent the main advantages of WFH: (i) work–life balance, (ii) improved work efficiency and (iii) greater work control. The main disadvantages were (iv) home office constraints, (v) work uncertainties and (vi) inadequate tools. Comparing gender, number of children at home, age and managers versus employees in relation to these factors provided insights into the differential impact of WFH on people’s lives. The factors help organisations understand where action is most needed to safeguard both performance and well-being. As the data were collected amidst the COVID-19 pandemic, we recommend further studies to validate the six factors and investigate their importance for well-being and performance in knowledge work.

## 1. Introduction

The radical increase in the number of people working from home (WFH) during 2020 has been one of the effects of the coronavirus (COVID-19) pandemic [1,2]. To lower the risk of spreading the virus, national governments across the world required people to WFH unless they were key workers [3,4]. Given this new situation, societies, organisations and workplaces across the world are now seeking “the new normal” (including the “future of work” and the “role of the office”) in which WFH is the norm and people can continue teleworking in pure or hybrid forms post-COVID-19 [5,6]. Given the potential for cost efficiencies in comparison to the traditional office and the aim of providing people with more flexibility in choosing where to work, organisations have announced their aim to reduce their office space and introduce blended home–office working conditions post-COVID-19. Insight into how people experience WFH is now even more important than before. Determining the main factors that constitute the experiences people have when WFH can provide insights into how to organise and manage distance work in the future. Given this unique situation, the aim of this study was to capture the immediate impact and provide insights into people’s experiences of WFH during the COVID-19 pandemic and to identify the main (groups of) advantages and disadvantages people experienced when WFH during the COVID-19 pandemic, with a particular focus on knowledge workers working across distances and cultures within the domain of intra-organisational distance work [7,8].

### 1.1. Distance Work, Telework and WFH

For decades, digital technologies have demanded–and forced–changes within organisations [9,10], which has also generated distance work [10,11], including telework, remote work and WFH. Distance work and management can occur at different locations, such as from home (telework), in satellite offices (intra-organisational work) or at customer or client locations (inter-organisational work) [7,8]. According to Fisher and Fisher, time, space and/or culture constitute the distance between managers and employees [1].

Previous studies have recognised the central issues in distance work, telework and WFH, including a whole range of possible advantages and disadvantages [12,13,14]. The advantages of distance work that have been mentioned are increased productivity, less stress, a better work–life balance [15], reduced commuting time [11,16], increased control of work patterns and being in less contact with others [17,18]. Conversely, scholars have also reported numerous potential disadvantages associated with distance work, including home-based telework, at an individual level [12,19]. These include isolation, misunderstandings, decreased interpersonal contact and role ambiguity [20,21]. Other studies have found that the work–life balance may also be challenged since boundaries become blurry, people work more hours, there can be a lack of support and visible leadership, and there may be less social interaction when isolated and detached from the workplace [22,23,24]. As telework involves the use of information and communication technologies, distance managers should provide access to appropriate technologies [24]. It is important to note that, according to several scholars [11,17], employees WFH have more positive and less negative job-related well-being experiences compared to days working in the office. Further, they emphasised the importance of recognising individual differences when adapting to teleworking environments [11].

Based on the literature, it is evident that several factors–both positive and negative–may be associated with the experiences of WFH and telework, and such factors need to be considered equal and independent. There is no consensus on which factors are the most important for whom, or how they combine to reflect people’s experiences of telework. Thus, the importance and relevance of these factors in the new circumstances of enforced WFH have not been determined. While we can assume that WFH brings a whole range of possible advantages and disadvantages to knowledge workers, it is unclear which factors are important in bringing about these advantages and disadvantages and how these could be grouped.

### 1.2. Knowledge Workers and WFH

Before the pandemic, it was mainly knowledge workers [25] who worked from home, typically for a small part of their working day [26]. In this study, the term “knowledge work” applies to work with non-material inputs and outputs, with the individuals as the primary bearers of knowledge (“pure” knowledge companies) [27]. Examples are workers who are typically employed in consulting companies, law firms and universities. This is in contrast to companies where knowledge is also embedded in a technology (i.e., high-tech companies), such as biotech [25]. A study of knowledge workers showed that they experience the same working conditions in different ways [28]. Consequently, it can be difficult to safeguard employee well-being and performance when working across distances or when managers are separated from employees by either time or geography [1]. However, managers can motivate and support the performance of their distance workers if they focus on the basic needs of their employees, such as working conditions and belongingness [29,30].

Given the unique situation that prompted governments to take the unprecedented step of forcing WFH on people (with the exception of key workers) and being amidst a pandemic, it could be anticipated that these circumstances would be extraordinarily stressful for people. Consequently, numerous national and international studies were initiated during 2020 that focused on the effects of the pandemic on people’s lives from various perspectives, including those who are WFH [31,32,33,34,35]. In practice, the emphasis has been on how to take care of people’s well-being and mental health when WFH [32,36]. There have been concurrent discussions regarding the experienced increase in productivity and work patterns and other potential gains attributable to WFH [37,38]. Accordingly, it is relevant to gain a deeper understanding of how people experience WFH and how they perceive the advantages and disadvantages of their new situation.

In this study, we investigated and analysed people’s experiences of WFH in the first months of lockdown and identified the key factors that could be distinguished based on these experiences. The remainder of this paper is divided into three sections. First, we present our study and the results relating to people’s experiences of WFH. We then present the key advantages and disadvantages of WFH, followed by examples of how the factors can be used to compare different demographic subgroups, which are displayed in a spider diagram. Finally, we discuss the implications for further research and the management of WFH in organisations.

## 2. Materials and Methods

### 2.1. Procedure

To capture the immediate impact of COVID-19 lockdowns on people’s lives, an online survey in Danish and English was published on social media platforms and disseminated via email from 21 March 2020. The survey included information on the study, the anonymity of the collected data, the future use of the data and the respondents’ right to delete their answers. The first question entailed the respondents confirming that they were over 18 years of age, had read all the information about the survey and were volunteering to participate. The sample was recruited by non-probabilistic snowball sampling because this exploratory study required rapid access to data during the COVID-19 lockdowns. Two weeks after Denmark entered lockdown, other countries (e.g., Sweden, Germany, the UK, the Netherlands, Spain and Italy) joined the study, and national data were collected in their respective languages using translations of the survey. The data reported in this study were collected until 11 May 2020.

### 2.2. Instruments

The survey was designed to address people’s experiences of WFH using several design criteria. First, we wanted to obtain information about people’s experiences of WFH via predefined categories and the possibility of open answers to allow the respondents to formulate their own understanding of the current situation. Second, the questions needed to be based on existing knowledge of distance work, management and the topics discussed in the media on the pandemic’s effect on people’s lives. Third, the survey needed to include household characteristics as the national lockdowns entailed the closure of day-care centres, kindergartens and primary and secondary schools. Fourth, the questionnaire needed to be able to be answered within approximately 10 min to minimise dropouts. Finally, the survey needed to be launched as quickly as possible to obtain the early experiences of people WFH. The lockdowns implied that the data would be collected online using a non-probability sampling methodology and that we would need to use our industry and academic networks and social media, with the consequence of sample bias by virtue of the responses mainly coming from people involved in knowledge work.

The survey was designed in Denmark during mid-March 2020 [39] and included 23 questions in six sections that covered topics related to the respondents’ experiences of the following:

1. Their current work situation
2. The advantages of WFH
3. The disadvantages of WFH
4. The use of digital work tools
5. Demographic information
6. Their overall life situation

This paper focuses particularly on the results of Section 2 and Section 3. For each of the 29 items relating to the advantages and disadvantages, the respondents indicated the extent to which they agreed that the advantage/disadvantage was applicable to their current setting using a five-point Likert scale: strongly disagree, disagree, neutral, agree and strongly agree.

Several items enquired about the known advantages of WFH, like control over work patterns (i.e., less time in meetings, fewer interruptions, more breaks (Q11b, d, e, i, k), catching up on work and being more productive (Q11c), better work–life balance (Q11f) and reduced commute time (Q11j). The items Q11g and Q11h focused on the working conditions when WFH (i.e., atmosphere and own food) and Q11m on the ease of contacting people and connecting. All three items were based on media discussions and the research team’s own first experiences of WFH during the first days of the lockdown. The questions related to the disadvantages of WFH (Q13) were devised in regards to the unique situation in combination with existing knowledge about telework (Q13a, b, c, n, o) and WFH. The items in the survey, hard to focus (Q13d), unsure of what to do (Q13e), missing food and the benefits of going to work (Q11f), and Q11g, Q11l, Q11m and Q11p were influenced by media discussions and the research team’s own first experiences of WFH during the first days of the lockdown. As schools and kindergartens were also locked down, we were interested in gaining insights into the effects of the new household conditions (i.e., disturbances and restrictions on getting out and being able to exercise). We were further interested to hear about its effects on tasks and jobs–was there enough to do, was it interesting, and could it be done with the provided data, documents, equipment and workplace (ergonomics at home) (Q11h, i, j, k, l, m, p)?

### 2.3. Respondents

This dataset included 5748 professional and managerial workers from Europe. The respondents came from 29 different countries, with the majority from Denmark (23.3%), Germany (23.1%), Italy (15.3%) and Sweden (14.5%). Most of the respondents were women (59.2%), and 75.2% possessed a university degree. Managers accounted for 23.0%, 34.6% had one or more children at home and the large majority (84.1%) only worked from home during the COVID-19 lockdown. Table 1 presents the respondents’ characteristics.

### 2.4. Data Analysis

All the analyses were conducted using IBM SPSS Statistics versions 24 and 27 (Armonk, NY, USA) and the descriptive statistics of the quantitative variables were reported as frequencies and percentages. The exploratory factor analyses [40] were performed with principal component analysis as the method to extract factors with eigenvalues >1, and both varimax and oblimin rotation types were employed for rotation to investigate how the items in the survey could be grouped into sets of advantages and disadvantages of WFH. Furthermore, we applied t-tests and analysis of variance (ANOVA) to test for significant differences between the different respondent groups regarding the advantages and disadvantages of WFH.

## 3. Results

### 3.1. Overview of Advantages and Disadvantages of WFH

The respondents rated 13 advantage and 16 disadvantage items on the Likert scale from 1 (strongly disagree) to 5 (strongly agree). Table 2 shows the items with their mean values and standard deviations (*SD*s). In addition to lowering the risk of contracting and spreading the disease, saving commuting time and greater flexibility (regarding food and breaks) were rated as the most important advantages. The biggest disadvantages were missing colleagues, missing getting out of the home and poor physical work conditions in the home office. What is also important to note in Table 2 is that for several items, there is considerable variance across individual knowledge workers. With a mid-point of 3 on the scale, one might consider any mean value above 3.5 as indicative of an experience that is commonly experienced as either an advantage or disadvantage. Notwithstanding, only a few of the items were in this range. Most of the items were on average in the middle with considerable variance around the mean: the WFH experience was found to be more individual than common across all the workers. This was an important finding in itself.

Our aim was to examine people’s experiences of WFH due to COVID-19, both positively and negatively. The items “I contribute to lowering the risk of spreading COVID-19” and “I do not expose myself to the risk of getting a disease” were excluded from further analyses. These items were related to the main reason for WFH (i.e., to prevent the further spread of COVID-19) rather than to how people were experiencing their current work setting.

**Figure 1:**
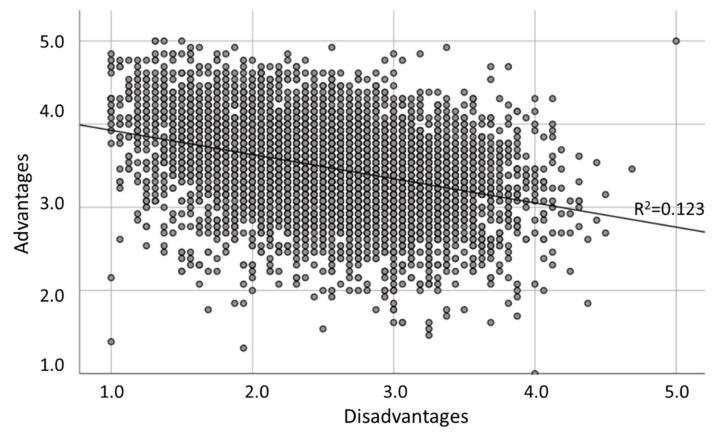
Positive (advantages) versus negative (disadvantages) working from home experience scores.

Using Cronbach’s alpha to analyse the internal consistency of the total sample, we found that the 11 remaining items relating to the advantages (Cronbach’s α = 0.739) and 16 items relating to the disadvantages (Cronbach’s α = 0.830) could be used to assign reliable scale scores to individual knowledge workers. Figure 1 shows a scatterplot of the advantages and disadvantages, where the middle point of the scales (neutral) lies at a score of three. The largest part of the point cloud is towards the top left, meaning the survey respondents reported relatively more positive and fewer negative experiences.

### 3.2. Finding the Key Factors in the WFH Experience

As mentioned, the 11 advantages and 16 disadvantages could be used as internally consistent scales to measure the levels of the advantages and disadvantages, respectively. The fact that the advantage and disadvantage items were sufficiently interrelated does not mean that the surveyed advantages and disadvantages all covered the same dimensions in the respondents’ experiences. The factor analyses pointed more in the direction of differentiation in the underlying factors; thus, it was considered more accurate to conduct analyses based on these items in terms of “item groups” of disadvantages and advantages rather than in terms of advantages and disadvantages in general. To identify specific factors in the advantage and disadvantage items, we split the original data set in two halves of 2874 cases each. For the first half of the data set, we performed explorative factor analysis (principal component analysis with varimax rotation (The oblimin rotation delivered almost exactly the same results. For reasons of parsimony, we have only reported the varimax-based solution here.)) and allocated items to the factor on which it loaded the highest. For the 11 items (with a five-point answering scale) relating to the advantages, three factors with eigenvalues >1 were found. Together, these three factors explained 49% of the variance. For the 16 items (with a five-point Likert scale) relating to the disadvantages, three factors with eigenvalues >1 were found. These three factors together explained 47% of the variance. For the test group with 2874 cases, we ran a factor analysis on each of the six proposed scales, forcing a one-factor solution. This allowed us to assess just how adequate it was to use factor analysis on these data, and how likely it is that information reduction can be achieved for the items in a scale. The results showed that the Kaiser–Meyer–Olkin value was always above 0.50, that Bartlett’s test of sphericity was highly significant (*p* < 0.001) and that the amount of variance explained by the single factor was always substantial, ranging from 36% to 62%. In addition, the exploratory factor analysis on the test group found exactly the same factor solution as for the development group.

Based on the results of the factor analysis, we derived three advantageous factors (AFs) and three disadvantageous factors (DFs), as shown in Table 3. The items were sorted according to the extent to which they determined the factor mentioned for the development group (using factor loading). We were able to construct scales that in 9 instances out of 16 were good by common standards (>0.70), while 4 of the 16 were already acceptable (0.60–0.70). The remaining four scales are not yet fully developed but provide useful starting material (alpha between 0.50 and 0.60). As mentioned, the seven scales with alphas below 0.70 required another run, with some modifications and some additions, building on the general factor structure that we have reported here. Good scales could be built from these materials for all six subscales. More attention is needed to improve the “scales” on AF1 and AF3.

### 3.3. How the Respondents’ Characteristics Related to the Advantages and Disadvantages

The six factors of the WFH experience can be used to analyse and compare different groups of workers using one-factor ANOVA (if more than two groups) or a *t*-test. When using ANOVA–and depending on the test of homogeneity of variances (Levene’s test), Bonferroni tests (as an example of a post hoc test) were also applied to find significant differences between the specific groups. In this section, we returned to using the full sample.

Table 4 presents the differences between the male and female knowledge workers in their perceptions of WFH. Although the women and men perceived the improved work–life balance in the same way, the female respondents perceived the home office constraints more than the male respondents. We found significant differences in AF 2 “Work efficiency”, DF 1 “Home office constraints” and DF 3 “Inadequate tools”. According to Table 4, the male respondents could work more effectively and efficiently from home; they felt less constrained by their home office and missed important work tools less than the women. Cohen’s *d* shows the medium to large effect sizes for the six factors.

Another interesting result was the difference between the people with children below 15 years of age at home (*N* = 1989) and the people without children at home (*N* = 3720). Missing values were excluded from the analysis (see Table 5).

The *t*-test revealed significant differences in all six factors with medium to large effect sizes. The biggest difference was visible in the AF 2 “Work efficiency” dimension, where the people without children scored higher than the people with children at home. This can be explained by the fact that most countries were in lockdown when the data were collected, meaning schools and kindergartens were closed and children were at home with their parents. Nevertheless, the people with children at home perceived the comfort of being at home as higher than the people without children, although they had less control over their working day. With regard to the disadvantages, the people with children at home felt the home office constraints more than the people without children. Moreover, the people without children scored higher in feeling the disadvantages because of an unclear work situation and the loss of important work tools.

With respect to age, Table 6 shows the differences between three age groups: 18–30 years (*N* = 749), 31–50 years (*N* = 3269) and >50 years (*N* = 1706). The people who preferred not to state their age were excluded from this analysis. The ANOVA indicated significant *F*-values (*p* = 0.000) for all six factors (Table 6) for the age classes, although the effect size was small.

Regarding the differences between the age groups, we used a Bonferroni post hoc test to investigate homogeneous subsets. The young people aged between 18 and 30 years scored the highest in AF 1 “Work–life balance” (mean = 3.6, *SD* = 0.77) compared to the people aged between 31 and 50 years (mean = 3.37, *SD* = 0.8) and the people over 50 years (mean = 3.35, *SD* = 0.76). On the other hand, the young people had more problems with “Work uncertainties” compared to the older generations. The people over 50 perceived “Inadequate tools” as a bigger problem (mean = 2.6, *SD* = 1.0) compared to the younger generations.

With regard to occupation, we compared managers (*N* = 1324) and employees (*N* = 4424). The *t*-test reported in Table 7 revealed significant differences between the two groups across all six factors. Cohen’s *d* shows the medium to high effect sizes.

Both groups scored higher for the advantages compared to the disadvantages. The employees evaluated their work efficiency and work–life balance more positively than the managers, whereas the managers reported fewer work uncertainties and less of a lack of important work tools compared to the employees.

**Figure 2:**
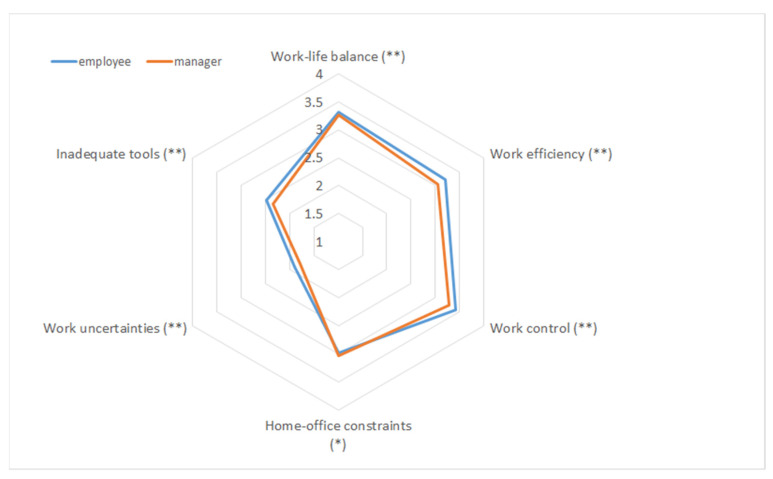
Visualising the differences in the factors between the employees and managers (* *p* < 0.05, ** *p* < 0.01).

The six factors of the WFH experiences were then used to analyse and compare the different groups exemplified and presented in Table 4, Table 5, Table 6 and Table 7. When the results were plotted in a spider diagram, the visualisation became a tool that made it possible to distinguish between the factors and compare the groups. Figure 2 is an example of a visualisation of Table 7. This tool can help clarify differences and help organisations understand where action is most needed in working conditions to ensure both performance and well-being.

## 4. Discussion

During the spring of 2020, the COVID-19 pandemic created an extraordinary situation for knowledge workers because of forced WFH as part of national lockdowns. The purpose of this study was to gain insights into the experiences of WFH among knowledge workers during the early weeks of the lockdowns and to determine the advantages and disadvantages of these experiences. Replies from 5748 professional and managerial workers from 29 European countries formed the data set for this study.

### 4.1. Overall Experiences of WFH during COVID-19

The first major topic we sought to address was the overall experience of WFH. The results revealed that WFH was experienced predominantly as positive for the majority of the respondents, with fewer respondents considering WFH mostly as a negative experience. Being in the middle of an international crisis and considering the pandemic’s potential for causing mental strain, we expected that the respondents would have experienced the situation of WFH during the national lockdowns as more negative [41]. However, in line with other recent studies, our findings showed that the majority (55%) of employees were mostly positive about WFH. These results may be due to the specific circumstances of the early lockdown, which granted an unanticipated opportunity to focus on work tasks and become more efficient (AF 2) and spend more time with people in the household (AF 1). Similarly, Pierce et al. (2020) found in a UK study that “for some people with high levels of socioeconomic security, the suspension of commuting, changes to education and work activities, and increased time with family potentially could have reduced stress and increased mental health and wellbeing” (p. 884) [31]. In a COVID-19 study in Italy, the participants also claimed that they were less stressed and equally satisfied compared to working in the office; however, they were less productive [42]. 

Accordingly, when organisations enter into discussions about how to proceed post-COVID-19 and meet with requests for increased usage of WFH compared to pre-COVID-19, they should understand the opportunities of WFH when deciding on a strategy as our results show that there are many positive experiences on which to build. However, one must also remember to accommodate those who are predominantly challenged by WFH (approximately 45% in our study) and try to gain insights into the various reasons for this. Recent COVID-19 studies have highlighted the risk of deteriorating mental health in different countries during the pandemic [31,43,44], which is disrupting mental health services [45]. This dualism in people’s experiences implies that organisations need to consider how people perceive WFH. The three advantages and three disadvantages outlined in this paper can act as pointers to identify which conditions affect which people.

### 4.2. The Six Factors

The second aim of this paper was to gain insights into the perceived advantages and disadvantages of WFH. The availability of a diverse sample allowed us to explore which factors constituted the experiences of WFH during the pandemic across different countries. In addition to lowering the risk of contracting and spreading the disease, the saving of commuting time and greater flexibility (regarding food and breaks) were rated as the most important advantages. The biggest disadvantages were missing colleagues, missing getting out of the home and poor physical work conditions in the home office. What is important to note from Table 2 is that, for many of the items, there was considerable variance across individual knowledge workers. With a mid-point of 3 on the scale, one might consider any mean value above 3.5 indicative of an experience that is commonly experienced as either an advantage or disadvantage. However, only a few of the items were in this range. Most of the items were on average in the middle with considerable variance around the mean. Accordingly, the WFH experience was found to be more individual than common across all the workers. This is an important finding in itself.

However, where previous studies on telework listed the advantages and disadvantages in a random order and/or focused on the single items in our list without relating their dependency, mutual importance or prioritisation, we conducted a factor analysis which showed that the different experiences were interrelated and could be grouped into six main factors. The six key advantages and disadvantages of WFH can be interpreted as the “common denominator” of how people experience WFH. This common denominator was derived from the existing literature as well as media accounts during the early stages of lockdown and grouped into main factors using the survey responses of more than 5000 knowledge workers. Such a “common denominator” is important as it provides a way to implement knowledge about how people experience WFH in practice and policy.

We perceive this finding as having two major implications. First, when organisations are planning to initiate or allow WFH to continue, they can focus on these six factors instead of all the potential advantages and disadvantages of WFH separately as the six factors provide the main areas in which to direct organisational efforts when considering new WFH practices. Additionally, the six factors may be used to monitor advantages and disadvantages when developing and implementing new strategies to improve working conditions and employee well-being.

### 4.3. Practical Implications of the Six Factors

In our view, the six factors are interesting in how they vary as a function of employee characteristics and roles. Using tests for mean differences to present mean scores in spider diagrams and evaluating and visualising the six factors using general characteristics, as for example with gender, having children at home, age group and role in the organisation, can lay the foundation for discussions about the current working conditions and help managers understand where action is needed. As European countries chose different strategies in their fight against COVID-19, it would also be relevant to know how these different approaches across countries might have contributed to workers’ experiences. It is beyond the scope of this paper to compare countries, but in our opinion, evaluating and visualising the six factors across countries could provide input into discussions regarding national strategies and the experiences of WFH.

With respect to WFH with children at home, our findings are consistent with the intense discussions about the effect of schools and kindergartens being in lockdown that has taken place in some countries [46,47]. Unsurprisingly, our analysis showed that people with children at home felt they were working less efficiently (AF 2) than the people with no children at home. Unexpectedly, the perceptions among managers versus employees showed that managers were more challenged than employees when WFH on some points but were better on others. In light of this result and the strong focus on maintaining mental health among employees during the pandemic, future work should also investigate managers’ specific situations during a pandemic and how they experience the transition towards becoming distance managers [48].

Our analysis indicated that “Inadequate tools” (DF 3) was one of the factors hindering the current way of working. As telework and WFH are likely to become much more commonplace post-COVID-19, organisations should consider how to ensure access to work tools and how to maintain the value of work to alleviate obstacles to efficiency when WFH, as also suggested by Eurofound [49].

In summarising the current status of an organisation and/or organisational subgroup with respect to the six factors, the visualisation in the spider diagram provides an easy tool to display the advantages and disadvantages inherent to the six factors for different groups of people (e.g., in different departments and teams). Furthermore, gender, age, occupation and/or further factor subgroups can be plotted and evaluated visually. Given the comparative analyses between the different groups, which provide a first insight into people’s perceptions of WFH, it would be interesting to analyse the concerns that have been addressed in public discussions across different demographic groups regarding parenting, home schooling, type of household, etc. This is beyond the scope of this study, but we see that the factors have the potential to provide deeper insights into people’s perceptions of WFH and the current state of a workplace if more demographic factors are included.

Such results can become part of a discussion in a company on how different employee groups perceive their WFH situation while transitioning from pre-COVID-19 via the coronavirus waves towards a post-COVID-19 stage. This provides a basis for developing concrete individual and organisational support for WFH as well as how to time such support per subgroup.

As the people in this study agreed with the statements that WFH increases their work efficiency, it would appear that there is something organisations can gain in terms of how they organise work post-COVID-19 once they start creating new ways of working. If people continue to work from home, by choice or because of future COVID-19 waves, the experiences presented in this study offer an opportunity to rethink WFH as well as distance management. The six factors can map out the directions for a balanced dialogue in which organisations can build on cultivating the advantages and minimising the disadvantages of WFH by focusing on these six factors in their decisions as they all constitute a relevant part of people’s experiences of WFH. We therefore see this study as the first step towards enhancing our understanding of the effect that WFH has on people by suggesting that organisations focus on the six factors. However, it is beyond the scope of this paper to analyse their implications, so further studies need to be performed to establish the relevance and meaning of the factors in an organisational setting.

### 4.4. WFH Post-COVID-19

Building on the positive experiences of WFH, more workplaces are likely to offer people the opportunity to continue WFH post-COVID-19 to meet the increased demand for flexibility [6,50,51]. Internationally, public and private companies have already taken the first steps in this direction [5,52], and the expectation is that it is possible to maintain high performance, support well-being and potentially reduce office space.

While there is a search for a new normal that includes the “future of work” and the “role of the office”, there is also an emerging concern about mental health and well-being outcomes [53,54,55]. While WFH has both advantages and disadvantages, as this study shows, it is important to note that these may affect people’s well-being and performance. This paper does not address this, but it is important for future research to examine how the six factors impact well-being and performance over time, not just for employees but also managers [48], and how to develop new ways to deliver organisational support to address this.

## 5. Limitations

While the strength of this study is that the data were collected in the first months of the pandemic, collecting data amidst a pandemic implies obvious limitations. First, applying snowball sampling to contact as many respondents as possible as we did includes the risk of bias in the data. Although the data were collected across 29 countries, the generalisation of our results only considers respondents with similar personal characteristics (i.e., knowledge workers). Currently the scales reported in this paper were only evaluated using an exploratory factor analysis and internal consistency. For further development of the scales, it is necessary to use confirmatory factor analysis (CFA) to test the psychometric quality of the scales more rigorously, in terms of factor structure as well as reliability.

While the three factors relating to disadvantages already meet common standards (alphas >0.70) for psychometric scales, the factors relating to advantages need some modifications and additions to achieve good psychometric scales by building on the items we developed in this study. We therefore propose that more national and international studies conducted with the same focus as this would provide an opportunity to validate the results of this study (including the six factors). Specifically, it would be important to replicate and improve the measures presented here in a post-COVID-19 setting before using them to monitor the adaptation of telework and WFH for individuals and organisations on a more structural basis after the pandemic. Finally, we focused solely on the insights that the factors gave between groups. Including more demographic factors could provide more insights into the experiences of WFH in distinct groups or situations like gender and parenthood as well as the utility of the factors. Future studies could thus contribute to a better understanding of the effects of WFH on people’s experiences and the distribution and importance of each factor.

## 6. Conclusions

The COVID-19 pandemic has radically changed the world of work and organisations. This quantitative study investigated knowledge worker’s experiences of WFH in 29 European countries during the early stages of the pandemic (i.e., mid-March to mid-May 2020) and, in particular, the advantages and disadvantages of WFH. In our study, the majority of the respondents in the European countries found their new work conditions to be mostly positive as they appreciated the advantages of WFH. Moreover, through factor analysis, we found that the advantages and disadvantages of telework and WFH clustered around six factors during the pandemic. We labelled the advantages (i) *work–life balance*, (ii) *work efficiency* and (iii) *work control* and the three disadvantages (iv) *home office constraints*, (v) *work uncertainties* and (vi) *inadequate tools*. Accordingly, while previous studies described many items relating to people’s experiences of WFH separately, our study showed that these experiences are interrelated and have a mutual order. Using spider diagrams, the six groups of advantages and disadvantages of WFH were visualised for different groups. Such spider diagrams can provide a basis for organisations to discuss, support and/or mitigate employees’ positive experiences and perceived challenges when WFH. As the research for this study was conducted in the early weeks of the pandemic, further studies are needed to examine the six factors across a longer time span and a wider range of types of workers. Such studies should also seek to determine whether the relatively positive experiences reported here will continue or whether these will change in a more negative direction over time as the lockdown continues and after the pandemic.

## Figures and Tables

**Table 1:** Characteristics of the respondents.

Variables	Total	Variables	Total
Gender, N (%)		Country, N (%)	
Female	3404 (59.2)	Austria	425 (7.4)
Male	2253 (39.2)	Belgium	26 (0.5)
Other	19 (0.3)	Denmark	1338 (23.3)
Prefer not to say	72 (1.3)	Finland	176 (3.1)
Age (years), N (%)		Germany	1328 (23.1)
18–20	5 (0.1)	Iceland	17 (0.3)
21–30	744 (12.9)	Italy	877 (15.3)
31–40	1561 (27.2)	Netherlands	259 (4.5)
41–50	1708 (29.7)	Norway	12 (0.2)
51–60	1357 (23.6)	Spain	286 (5.0)
>60	349 (6.1)	Sweden	834 (14.5)
Prefer not to say	24 (0.4)	Switzerland	24 (0.4)
Highest level of education, N (%)		United Kingdom	108 (1.9)
No schooling completed	1 (0.0)	Other European countries	36 (0.6)
Primary education	22 (0.4)	No. of children younger than 15 at home, N (%)	
Secondary education	142 (2.5)	0	3720 (64.7)
Vocational training	392 (6.8)	1	931 (16.2)
Associate degree	623 (10.8)	2	898 (15.6)
Bachelor’s degree	820 (14.3)	3	160 (2.8)
Master’s degree	2585 (45.0)	missing	39 (0.7)
Doctorate degree	916 (15.9)	No. of people including yourself at home, N (%)	
Other not listed degree	215 (2.7)	1	1578 (27.5)
Kind of work, multiple answers possible, N (%)		2	1861 (32.4)
Administrative work	2207 (38.4)	3	1017 (17.7)
Research	1187 (20.7)	4	938 (16.3)
Develop systems, plans	1048 (18.2)	5 or more	290 (5.0)
Manager	1324 (23.0)	Missing	64 (1.1)
Communication	671 (11.7)	Work from home since COVID-19, N (%)	
Teaching/supervision	1028 (17.9)	Only work from home	4835 (84.1)
Marketing and sales	326 (5.7)	Sometimes from home	913 (15.9)
System monitoring	376 (6.5)		
Manufacturing	245 (4.3)		
Creative production	242 (4.2)		

**Table 2:** Overview of advantages and disadvantages of WFH.

Variables	Mean Value	SD
**Advantages**		
A: I contribute to lowering the risk of spreading COVID-19.	4.60	0.72
B: I get time to focus on my work without interruptions from other people.	3.48	1.23
C: It is possible for me to do some other work that I would normally not have time to do.	3.05	1.21
D: I do not have to spend time in long meetings.	2.98	1.21
E: I can take a break when I want to.	3.66	1.08
F: I can be close to my family and friends.	3.41	1.23
G: I like the atmosphere in my home better than at work.	2.93	1.07
H: I can eat and drink my own food.	3.80	1.06
I: I have no one looking over me.	2.73	1.13
J: I save on the normal commute time to my workplace.	4.37	0.94
K: I do not expose myself to the risk of getting a disease.	4.38	0.81
L: I have a chance to break my old habits and change my routines.	3.29	1.09
M: It is easier to get in contact with people than normal.	2.52	1.06
**Disadvantages**		
A: I do not get to see my colleagues or other people as much as I would like to.	3.83	1.12
B: I need physical equipment that I do not have access to at home to do my work.	2.71	1.30
C: I need data or documents that I do not have access to at home to do my work.	2.21	1.20
D: I find it difficult to keep focused on my work when I am alone.	2.04	1.14
E: I don’t know what kind of work I should do.	1.58	0.86
F: I miss the food or other benefits that we have at my workplace.	2.19	1.24
G: It is a financial problem for my work that I cannot be at the workplace.	1.73	0.96
H: I get disturbed by other people in my home.	2.52	1.36
I: I miss getting out of my home.	3.67	1.24
J: I do not get enough exercise when I am not at my workplace.	3.06	1.37
K: The work I do from home is not as interesting as the work I do at my workplace.	2.19	1.18
L: I am afraid that there will not be enough work for me to do from home.	1.88	1.08
M: The physical conditions in my home do not afford a good working environment (adjustable table and chair, enough light, quietness, good monitor, etc.).	3.06	1.42
N: It requires more effort from me because I cannot use my normal routines.	2.52	1.21
O: I feel tied to my computer to a greater extent than when at my workplace.	3.11	1.34
P: I am concerned that there are work tasks I want to do but cannot do from home.	2.44	1.26

**Table 3:** Results of the exploratory factor analysis (varimax rotation).

Factor Description	Factor/Items	Factor Loading (Development Group)	Factor Loading (Test Group)	Cronbach’s Alpha (Development Group)	Cronbach’s Alpha (Test Group)
**Clusters of advantages of working from home**					
AF 1: Work–life balance Instead of going to work and wasting commute time, you can enjoy the atmosphere at home, change routines and have more time for your social life.	G: I like the atmosphere in my home better…	0.650	0.598	0.59	0.58
	J: I save on the normal commute time…	0.617	0.559		
	M: It is easier to get in contact with people…	0.592	0.659		
	L: I break my old habits and change my routines.	0.525	0.577		
	F: I can be close to my family and friends.	0.374	0.413		
AF 2: Work efficiency Instead of spending time on meetings and wasting time on meaningless tasks at work, you can focus on your tasks without interruptions.	D: I do not have to spend time in long meetings…	0.717	0.710		
	
	C: It is possible for me to do some other work… B: I have time to focus on my work …	0.702 0.600	0.721 0.681	0.61	0.62
AF 3: Work control Instead of being controlled, you can take a break when you want and have more control over your day.	I: I have no one looking over me.	0.775	0.730		
	E: I can take a break when I want.	0.609	0.616	0.53	0.55
	H: I can eat and drink my own food.	0.532	0.637		
**Clusters of disadvantages of working from home**					
DF 1: Home office constraints Instead of a life with social interaction and exercise, you have limited contact with people, get out of the home less, are more fixed in front of the computer and get disturbed by others at home.	I: I miss getting out of my home.	0.683	0.715		
	A: I do not get to see my colleagues … as much…	0.614	0.647		
	J: I do not get enough exercise…	0.598	0.646		
	M: The physical conditions in my home do not afford…	0.596	0.543	0.73	0.74
	F: I miss the food or other benefits … at my workplace.	0.517	0.512		
	N: It requires more effort from me that I cannot use my normal routines.	0.516	0.503		
	O: I feel tied to my computer to a greater extent …	0.475	0.494		
	H: I get disturbed by other people in my home.	0.473	0.432		
DF 2: Work uncertainties Instead of finding meaning in work, the work situation is unclear as there is not enough to do, the remaining tasks are not interesting, financial problems might occur and you cannot focus on your work.	L: I am afraid that there will not be enough work…	0.750	0.789		
	
	E: I don’t know what kind of work I should do.	0.712	0.715		
	K: The work I do from home is not as interesting …	0.648	0.684	0.71	0.72
	G: It is a financial problem for my work…	0.548	0.489		
	D: I find it difficult to keep focused on my work…	0.502	0.512		
DF 3: Inadequate tools Instead of having easy access to what you need to perform your work, you lose the valuable work tools, data and documents required to do your work adequately.	B: I need physical equipment … to do my work.	0.822	0.784		
	
	C: I need data or documents … to do my work.	0.778	0.782	0.71	0.69
	P: …there are work tasks I… cannot do from home.	0.570	0.567		

**Table 4:** Mean values, standard deviations and *t*-test results for gender.

	Women, Mean (*SD*)	Men, Mean (*SD*)	*t*-Value	*p*-Value	Cohen’s *d*
AF 1: Work–life balance	3.30 (0.67)	3.31 (0.65)	−0.250	0.802	0.66231
AF 2: Work efficiency	3.12 (0.93)	3.24 (0.88)	−4.644	0.000 **	0.91149
AF 3: Work control	3.39 (0.79)	3.39 (0.78)	−0.179	0.858	0.78800
DF 1: Home office constraints	3.07 (0.77)	2.90 (0.75)	8.197	0.000 **	0.76107
DF 2: Work uncertainties	1.88 (0.73)	1.90 (0.71)	−1.051	0.293	0.71963
DF 3: Inadequate tools	2.49 (0.99)	2.39 (0.99)	3.725	0.000 **	0.98910

** *p* < 0.01; SD standard deviation.

**Table 5:** Results of the *t*-test for the number of children <15 years at home.

	No Children, Mean (*SD*)	Children at Home, Mean (*SD*)	*t*-Value	*p*-Value	Cohen’s *d*
AF 1: Work–life balance	3.25 (0.67)	3.4 (0.64)	−7.951	0.000 **	0.66035
AF 2: Work efficiency	3.29 (0.87)	2.95 (0.96)	13.206	0.000 **	0.90018
AF 3: Work control	3.43 (0.79)	3.32 (0.78)	5.352	0.000 **	0.78561
DF 1: Home office constraints	2.92 (0.75)	3.13 (0.79)	−10.133	0.000 **	0.7593
DF 2: Work uncertainties	1.90 (0.74)	1.84 (0.68)	3.156	0.002 **	0.71886
DF 3: Inadequate tools	2.48 (1.00)	2.41 (0.97)	2.619	0.009 **	0.99027

** *p* < 0.01; SD standard deviation.

**Table 6:** ANOVA results for the different age classes.

	18–30 Years, Mean (*SD*)	31–50 Years, Mean (*SD*)	>50 Years, Mean (*SD*)	*F*-Value	*p*-Value	Partial Eta-Squared
AF 1: Work–life balance	3.22 (0.71)	3.35 (0.67)	3.26 (0.62)	18.711	0.000 **	0.007
AF 2: Work efficiency	3.23 (0.9)	3.11 (0.95)	3.25 (0.84)	15.850	0.000 **	0.006
AF 3: Work control	3.60 (0.77)	3.37 (0.80)	3.35 (0.76)	30.658	0.000 **	0.011
DF 1: Home office constraints	3.07 (0.75)	3.02 (0.79)	2.92 (0.73)	14.413	0.000 **	0.005
DF 2: Work uncertainties	2.95 (0.78)	1.84 (0.70)	1.9 (0.72)	26.440	0.000 **	0.009
DF 3: Inadequate tools	2.37 (0.98)	2.39 (0.98)	2.6 (1.00)	28.269	0.000 **	0.010

** *p* < 0.01; *SD* standard deviation.

**Table 7:** Results of the *t*-tests for employees and managers.

	Employees, Mean (*SD*)	Managers, Mean (*SD*)	*t*-Value	*p*-Value	Cohen’s *d*
AF 1: Work–life balance	3.32 (0.67)	3.26 (0.63)	3.040	0.002 **	0.66360
AF 2: Work efficiency	3.21 (0.91)	3.05 (0.93)	5.573	0.000 **	0.91271
AF 3: Work control	3.43 (0.78)	3.28 (0.79)	5.889	0.000 **	0.78508
DF 1: Home office constraints	2.98 (0.77)	3.04 (0.74)	−2.519	0.012 **	0.76577
DF 2: Work uncertainties	1.91 (0.73)	1.79 (0.66)	6.018	0.000 **	0.71726
DF 3: Inadequate tools	2.49 (1.00)	2.34 (0.93)	4.939	0.000 **	0.99046

** *p* < 0.01; SD standard deviation.

## Data Availability

The data presented in this study is not publicly available due to ongoing analysis but are available from the corresponding author on reasonable request.
